# Multiple myeloma in the real world settings: prognostic significance of 1q21 chromosomal abnormalities - single center experience

**DOI:** 10.3389/fonc.2026.1765235

**Published:** 2026-02-11

**Authors:** Aleksandra Sretenovic, Marko Mitrovic, Zoran Bukumiric, Nikola Vukosavljevic, Natalija Kecman, Jelica Jovanovic, Marija Dencic Fekete, Enisa Zaric, Jelena Bila

**Affiliations:** 1 Clinic of Hematology, University Clinical Center of Serbia, Belgrade, Serbia; 2 Medical Faculty, University of Belgrade, Belgrade, Serbia; 3 Institute of Statistics and Bioinformatics, Medical Faculty, University of Belgrade, Belgrade, Serbia; 4 University Clinical Hospital Center “Zvezdara”, Department for Hemato-Oncology, Belgrade, Serbia; 5 Institute of Pathology, Medical Faculty, University of Belgrade, Belgrade, Serbia; 6 Department of Hematology, University Clinical Center of Podgorica, Medical Faculty, University of Podgorica, Podgorica, Montenegro

**Keywords:** 1q21 chromosomal abnormalities, multiple myeloma, prognosis, risk stratification, treatment

## Abstract

**Background:**

Although prognostic significance of 1q21 chromosomal abnormalities (CAs) in multiple myeloma (MM) has been validated, the real world data (RWD) of its practical utilization in the clinical practice remain to be of interest. The aim of study was to analyze the prognostic significance of 1q21 CAs in the RWD settings of the routine clinical practice.

**Methods:**

The study included 328 newly diagnosed MM patients (NDMM, pts), diagnosed during the period 2018-2024. The distribution according to the second Revision of the International Staging System (R2-ISS) was 25.6%, 22.9%, 46.3% and 5.2% with scores 1, 2, 3 and 4, respectively. The 1q21 CAs, gain1q21 and amp1q21, were detected in 33.7% pts (gain1q21 in 12.4% pts, and amp1q21 in 21.3% pts). Patients were treated predominantly (238pts, 72.6%) with Bortezomib (Bz) plus immunomodulatory drugs (IMids) based triplets. Autologous stem cell transplantation (ASCT) were performed in 87/114 potential transplant candidates (76.3%).

**Results:**

The overall response rate (ORR ≥PR) was achieved in 86.3% of the patients, regardless of the findings of gain 1q21 (p=0.113) or amp1q21 (p=0.757) in comparison to the patients without 1q21 CAs. Patients without 1q21 CAs had significantly longer progression-free survival in comparison to the patients with gain 1q21 or amp1q21 (PFS, p=0.000). The overall survival of the patients with amp1q21 was significantly shorter in comparison to the patients with gain1q21 or those without 1q21 CAs (OS, p=0.043).

**Conclusion:**

Presence of the 1q21 CAs retains clear impact on the course of disease in MM patients outside of clinical trials. As a part of validated prognostic indices, finding of 1q21 CAs represents valuable prognostic biomarker, which may contribute in bringing up treatment choice, especially in the circumstances of limited accessibility to the new treatment modalities.

## 1 Introduction

Multiple myeloma (MM) is a complex disease, characterized by a distinct clinical heterogeneity in the response rate and survival outcomes (1). Despite the significant improvements in the treatment outcome and overall survival (OS), most of multiple myeloma patients (pts) eventually relapse and develop refractory disease (1).

Based on high degree of MM genomic instability and findings of frequent numerical and structural chromosomal aberrations associated with prognostic impact on the course of disease and overall survival of MM pts, different prognostic models have been validated and established during course of the time in addition to a historical varieties of classical clinical and laboratory parameters that are an integral part of prognostic models (2, 3).

One of the most common numerical chromosomal aberrations in multiple myeloma are abnormalities of chromosome 1q21, presented as a gain1q21 or amp1q21 depending of the number of copies, three or ≥4 copies respectively, occurring in 30-40% of newly diagnosed MM (NDMM) pts. The frequency of 1q21 CAs raises during the course of disease up to 80% in relapsed-refractory MM (RRMM) pts (4, 5). Patients with 1q21 CAs are considered to be of high risk, expressing multidrug resistance, with poor treatment outcome (5, 6). Yet, although prognostic significance of 1q21 CAs in multiple has been validated, the real world data (RWD) of its utilization in the everyday clinical practice with impact on the treatment decision, remain to be of interest (7, 8).

The aim of study was to analyze the prognostic significance of 1q21 CAs findings of gain1q21 or amp1q21 in NDMM pts diagnosed and treated within the RWD settings of the routine clinical practice.

## 2 Materials and methods

### 2.1 Patients

This retrospective study included 328 NDMM patients, diagnosed and treated from January 2018 to January 2024 at the Department for Multiple Myeloma, Clinic of Hematology University Clinical Center of Serbia.

The median age was 67 years (range 34-90yrs), with slight predominance of female pts (male 163pts, 49.7%; female 165pts, 50.3%). The distribution according to the type of MM was as follows: IgG myeloma was diagnosed in 204pts (62.2%); IgA in 69pts (21%); light chains in 54pts (16.5%); and IgD in 1pts (0.3%). Of 328pts, 276pts (84.1%) had advanced clinical stage (CS) III according to the Durie&Salmon staging system, 28pts (8.6%) had CS II; and 24pts (7.3%) had symptomatic CS I (9). Renal impairment according to the criteria of International Myeloma Working Group (IMWG) was present in 78pts (23.9%) (10). Stratification of the MM pts, using International Staging System (ISS), was: 99pts (30.2%) had score 1; 88pts (26.8%) an ISS score 2; and 141pts (43%) an ISS score of 3 (11). After establishing the MM diagnosis, specific chromosomal abnormalities (CAs: t(4;14); del(17p); t(14;16), +1q21; amp1q21) were examined at the smears of plasma cells isolated from the bone marrow aspirates and purified with CD138 microbeads with purity level of 90%. Interphase fluorescence *in-situ* hybridization (iFISH) as previously described, according to ESMO, EHA-ESMO and EHA-EMN Guidelines for diagnosis, treatment and follow-up (7, 8, 12–14). The minimum number of nuclei scored was 200 per case. Gain of chromosome 1q21 was defined as the presence of three copies of the 1q21 locus in at least 20% of analyzed nuclei, while amplification was defined as four or more copies of the 1q21 locus with the same positivity threshold (at least 20% of nuclei). The risk stratification of MM pts was conducted using Revised ISS (R-ISS), and second Revision of ISS (R2-ISS) staging system (15–17). The distribution according to the R-ISS and R2-ISS scores was as follows: 92pts (28%) had R-ISS 1 vs. 84pts (25.6%) with R2-ISS 1; 201pts (61.3%) had R-ISS 2 vs. R2-ISS 2 75pts (22.9%); while 35pts (10.7%) had R-ISS 3 vs. 152pts (46.3%) had R2-ISS 3 and 17pts (5.2%) with R2-ISS 4. The patients’ clinical characteristics are summarized in **Supplementary Table S1**.

### 2.2 Treatment

Treatment with bortezomib (Bz) plus immunomodulatory (IMids) based triplet combinations was applied in 238 patients (72.6%), while high-dose treatment and autologous stem cell transplantation (ASCT) was performed 87pts of 114 potential transplant candidates (76.3%). The treatment choice was based on the pts characteristics: 1) age ≤70 years and transplant eligibility; 2) risk stratification in accordance to the R-ISS and R2-ISS score, presence of double-triple hit myeloma; and 3) simplified IMWG frailty score (12–14, 18). Tandem ASCT was performed in 27 of 87pts (31%) with high-risk features as R-ISS 3, or R2-ISS 3 and 4, or double/triple-hit MM. The treatment of the 78 frail pts (32.7%) defined with simplified IMWG score ≥2 were initiated with Bz-based doublets, and escalated treatment to Bz+IMids triplets in the case of recovery to intermediate-fit with simplified IMWG score 1.

Treatment response was evaluated according to IMWG criteria for the treatment response of pts with multiple myeloma (19).

With provision of informed consent, the pts clinical data were collected. The study was performed according to the guidelines of the Declaration of Helsinki Principles and Good Clinical Practice, and approved by the Institutional Board of the Hematology Clinic and Ethics Committee of the University Clinical Center of Serbia.

### 2.3 Statistical analysis

The clinical characteristics of pts were analyzed using descriptive statistics. Statistical analysis included the following testing methods: t-test, Mann-Whitney test, chi-square test and Fisher’s exact probability test. The Fisher’s exact test was used to analyze the categorical variables. The Kaplan-Meier method and log-rank statistics were used to determine the differences in OS and progression-free survival (PFS) of analyzed groups. Furthermore, a Cox regression model was used to determine which factors had the most significant effect on PFS and OS in MM patients. Statistical significance was considered present at p ≤ 0.05. All calculations were conducted using IBM SPSS Statistics version 22.0 (R software environment version 4.3.0; R Core team 2023).

## 4 Results

### 3.1 iFISH findings and treatment response

Chromosomal abnormalities (CAs) of the 1q21 region were detected in 76 of 225 pts with evaluable FISH findings (33.7%) obtained at diagnosis: gain1q21 was present in 28 (12.4%) and amp1q21 in 48 patients (21.3%). Double and triple-hit (DTH) MM was observed in 16 patients (5.6%). (**Supplementary Table S2**.) In 103/328pts (31.4%), main reasons for missing iFISH findings (≈17pts per year, 30.9% annually) were: poor performance status of patient (47pts, 45.6%); personal attitude of patient (29pts, 28.2%); and different technical limitations e.g. inadequate sampling and/or shipping and/or technically non-interpretive smears (27pts, 26.2%).

Overall response rate (ORR, ≥PR) was achieved in 273 pts (86.3%). There was no significant difference in ORR between pts with gain1q21 (Pearson Chi-Square 2.515, p=0.113) or amp1q21 (Pearson Chi-Square 0.096, p=0.757) in comparison to the pts without 1q21 CAs.

### 3.2 Survival analysis

**Figure 1:**
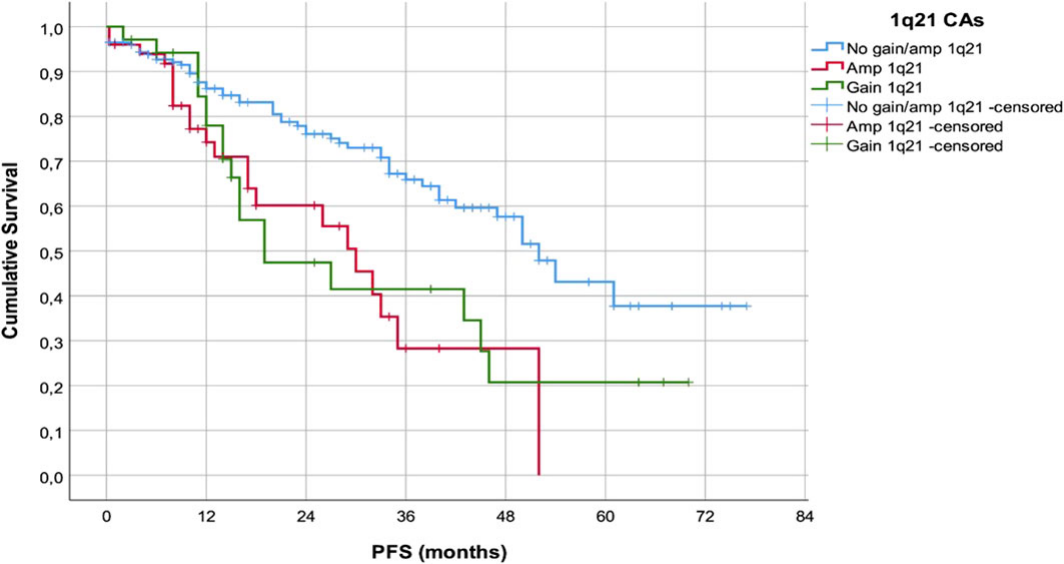
PFS of the patients in accordance with the presence of 1q21 CAs.

With median follow-up of 20 m (range 6–78 m), pts without 1q21 CAs had significantly longer PFS (median 52.5m, range 45.6-58.4m) in comparison to the pts with gain1q21 or amp1q21 (median 21.2m, range 4.7-37.6m, Log Rank 15.963, p=0.000) (**Figure 1**).

**Figure 2:**
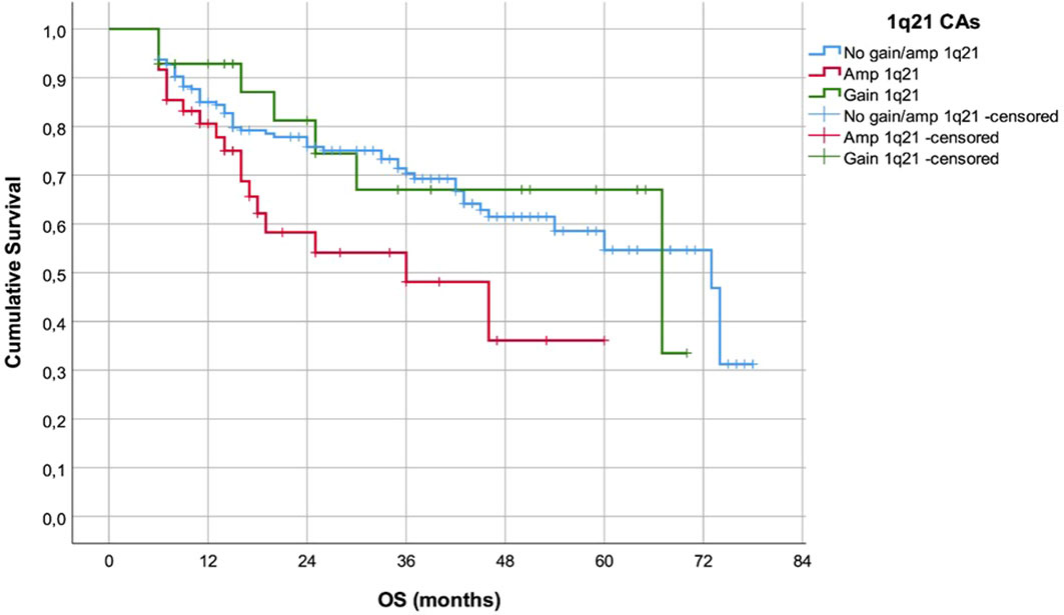
OS of the patients in accordance with the presence of 1q21 CAs.

Patients with amp1q21 had significantly shorter estimated OS (median 36m, range 13-59.0m) in comparison to the pts with gain1q21 or without 1q21 CAs (median 67m, range 14.2-119.8m, Log Rank 6.310, p=0.043) (**Figure 2**).

**Figure 3:**
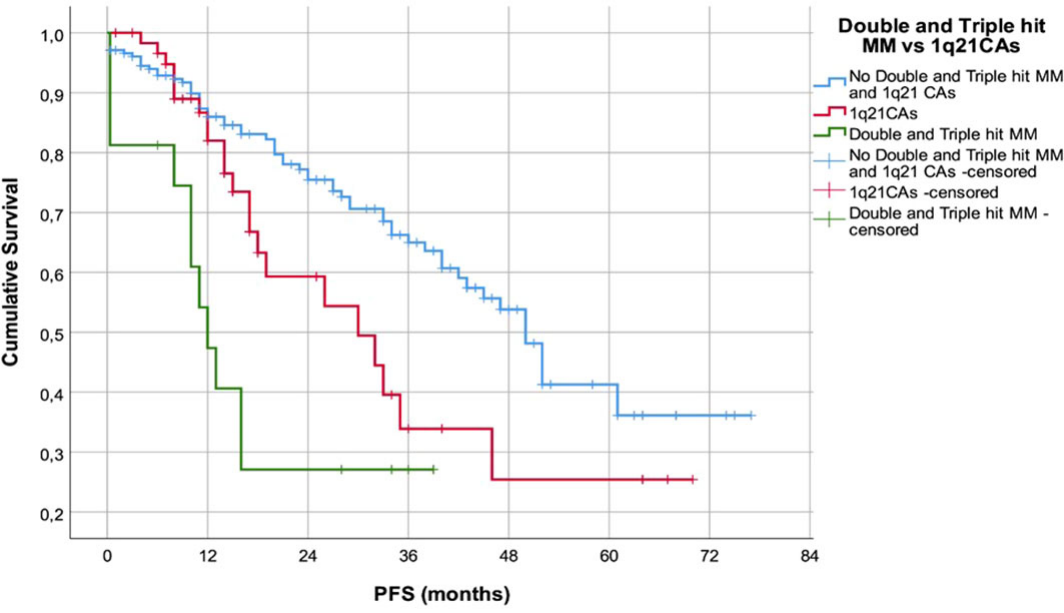
PFS of the patients with 1q21 CAs in comparison to patients with DHT hit MM.

The PFS of pts with double and triple hit (DHT) MM was significantly shorter (median 12m, range 8.4-37.6m) in comparison to the patients with 1q21 CAs (median 35.7m, range 13.7-70.8m, Log Rank 20.198, p=0.000) (**Figure 3**.).

**Figure 4:**
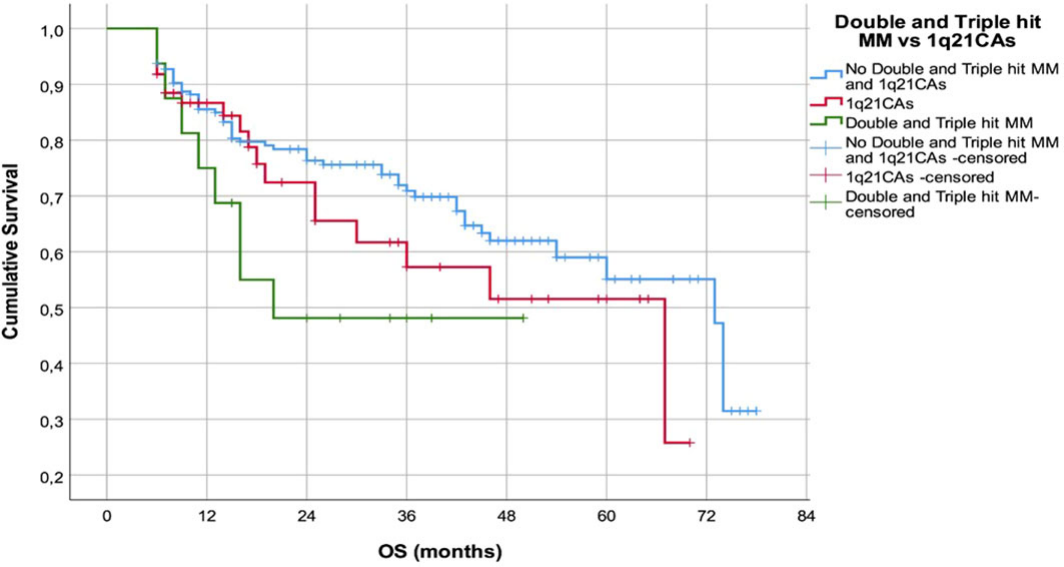
OS of the patients with 1q21 CAs in comparison to patients with DHT hit MM.

This finding was accompanied with shorter OS of pts with DHT MM (median 20.6m, range 5.7-49.1m) but without statistical difference in comparison to the pts with 1q21CAs (Log Rank 4.895, p=0.087) (**Figure 4**.).

The multivariate COX analysis pointed out prognostic impact on the PFS of following variables: ASCT eligibility (p=0.001; HR 0.39, 95% CI 0.20-0.64); R2-ISS scoring system (p=0.001; HR 1.39, 95% CI 1.22-2.16); while amp1q21 statistically was not significant in multivariate analysis (p=0.058; HR 1.68, 95% CI 0.98-2.88). Continuing this trend, the OS was mostly influenced by ASCT eligibility (p=0.000; HR 0.17, 95% CI 0.08-0.35); and R2-ISS scoring system (p=0.000; HR 1.7, 95% CI 1.23-2.20), (**Supplementary Table S3**).

## 5 Discussion

Chromosomal abnormalities involving the 1q21 region represent one of the most frequent genomic events in MM and are consistently associated with adverse outcomes across disease stages (4–6, 20–26). In our real-world cohort, 1q21 chromosomal abnormalities were identified in 33.7% of tested pts, with gain1q21 present in 12.4% and amp1q21 in 21.3%, consistent with previously published data reporting +1q21 in 30–40% of NDMM and up to 70% of RRMM (4, 6, 20, 23). Previous large genomic series, including CoMMpass, HOVON, and Mayo Clinic cohorts, demonstrated similar prevalence and clinical relevance of 1q21 copy-number alterations (20, 27–31). The frequency of DHT myeloma in our study (5.6%) also aligns with estimates of 5–8% in genomic analyses evaluating co-occurrence of del17p, t(4;14), t(14;16), del1p, and 1q amplification (24, 27, 32). In the RWD settings, observed frequency of 1q21 CAs is in accordance to the published data, despite the fact that 31.4% of patients from our group are missing the iFISH findings at diagnosis which mirrors the real-life situations of the routine clinical practice (7, 8).

In accordance to the international recommendations for diagnostics and treatment of MM at the time of diagnosis, the patients were treated with Bz+ImiDs triplets in the first line of treatment. However, 32.7% frail pts with simplified IMWG score ≥2 were initially treated with Bz-based doublets, and escalated treatment to Bz+IMids triplets in the case of recovery (12, 13). Regarding treatment response, in analyzed group 1q21 CAs did not significantly influence the ORR, consistent with major datasets such as IFM/DFCI 2009, EMN02/HO95, and CoMMpass, all of which demonstrated preserved early treatment sensitivity despite poor long-term outcomes (20, 32, 33). However, several studies have reported inferior ORR with bortezomib-based regimens in 1q21+ disease, particularly in RRMM, suggesting importance of the applied treatment specificity (20, 21).

Furthermore, above cited analyses indicate that +1q21 and amp1q21 primarily impact the course of disease rather than initial treatment response. In accordance, survival analyses in our cohort confirmed a significant adverse prognostic role of 1q21 abnormalities. Patients with gain1q21 or amp1q21 had shorter PFS compared with those without 1q21 alterations (p<0.001), consistent with previous large cohort analyses identifying 1q21 as a major driver of early relapse (7, 8, 17, 18, 20, 21). Moreover, pts with amp1q21 exhibited inferior OS compared with gain1q21 and 1q-negative pts(p=0.043), supporting the significance of 1q21 copy-number, a finding repeatedly demonstrated in prior studies (5, 20, 21). Amplification (≥4 copies) is biologically associated with increased expression of oncogenic regulators such as CKS1B and MCL1 (4, 5, 23).

Patients with DTH MM had significantly shorter PFS compared with isolated 1q21 abnormality (p<0.001), and duration of their OS was shorter (p=0.087). These findings mirror data from different cohorts indicating that combinations of high-risk chromosomal abnormalities with 1q21 CAs define an ultra-high-risk group with extremely poor outcomes (21, 28, 29). Furthermore, modern staging frameworks (R-ISS and R2-ISS) demonstrated expected risk redistribution in our cohort, particularly due to incorporation of 1q21 copy-number status in R2-ISS. Applying R2-ISS stratification, pointed out higher number of our patients with R2-ISS 3 and 4 groups, in align with findings of the Cox multivariate analysis and strong impact of the R2-ISS score on the PFS (p=0.001; HR 1.39, 95% CI 1.22-2.16), and OS (p=0.000; HR 1.7, 95% CI 1.23-2.20), which is consistent with EMN/HARMONY results validating R2-ISS as a superior prognostic tool (15, 21).

The most recent IMWG high-risk consensus (2024–2025) emphasizes integration of clinical and cytogenetic risk factors. Gain/amp1q21, del17p, t(4;14), t(14;16), and del1p represent the core genomic lesions defining high-risk MM with amp1q21 as one of the strongest predictors of early progression. Along with R2-ISS scoring system, those findings are indicating the limited significance of 1q21 CAs as independent prognostic factor. In the RWD settings, Next Generation Sequencing (NGS)-based methodology which offers enhanced detection of high-risk subclones and more sensitive MRD assessment compared with iFISH, is still in process of surpassing the difficulties of adequately incorporating and interpreting results, apart from significant financial burden of the method (33, 34).

Therapeutically, management of 1q21+ MM remains challenging. Evidence regarding proteasome inhibitors supports limited ability of Bz to overcome the adverse effect of amp1q21 (20, 21), whereas carfilzomib-based induction (KRd) has demonstrated improved PFS and MRD negativity in high-risk subsets (35). However, in the RWD circumstances characterized with limited access to the new treatment modalities outside of clinical trials, in analyzed group of pts, tandem ASCT was conducted in high-risk transplant eligible patients, which was in accordance with international recommendations at the time of diagnosis, and CIBMTR findings that intensification with high-dose treatment and ASCT may partly reduce, but not eliminate, the adverse prognostic effect of 1q21 abnormalities (12–14, 36).

Currently, monoclonal antibodies, particularly daratumumab, have improved outcomes in high-risk subgroups, though evidence in 1q21+ disease remains limited (37). In this context, the PERSEUS and CEPHEUS phase III trials established daratumumab-VRd (D-VRd) quadruplets as a new standard in the frontline setting for transplant-eligible and transplant-ineligible, overcoming the inferior outcome of the high-risk pts including those with 1q21 CAs (38, 39). Beyond daratumumab, isatuximab has shown as well significant benefit in the frontline settings of the treatment high-risk patients with 1q21 CAs (40, 41).

Novel immunotherapies, including CAR-T cells and bispecific antibodies, proved their efficacy in RRMM settings, including high-risk disease (13, 14). Future real-world analysis of the growing clinical experience in accordance to the current guidelines and recommendations, are needed in defining the optimal sequencing of the new immuno- and cellular therapies, especially in the high-risk population of pts including pts with gain/amp(1q21) (42, 43).

## 6 Conclusion

Our findings confirm the prognostic impact of the 1q21 CAs, in particular amp1q21, on the course of disease and outcome of the pts with MM in the RWD settings. However, the significance of 1q21 CAs as independent prognostic factor is still matter of debates. Early identification of 1q21 CAs, along with other high-risk chromosomal abnormalities, or association with DTH, is mandatory for the optimal treatment decision which should be based on the validated prognostic scores as R2-ISS and IMWG high-risk consensus, especially in the circumstances of limited accessibility to the new treatment modalities.

## Data Availability

The raw data supporting the conclusions of this article will be made available by the authors, without undue reservation.
