# Correction: Multiple myeloma in the real world settings: prognostic significance of 1q21 chromosomal abnormalities - single center experience

**DOI:** 10.3389/fonc.2026.1920779

**Published:** 2026-07-21

**Authors:** Aleksandra Sretenovic, Marko Mitrovic, Zoran Bukumiric, Nikola Vukosavljevic, Natalija Kecman, Jelica Jovanovic, Marija Dencic Fekete, Enisa Zaric, Jelena Bila

**Affiliations:** 1 Clinic of Hematology, University Clinical Center of Serbia, Belgrade, Serbia; 2 Medical Faculty, University of Belgrade, Belgrade, Serbia; 3 Institute of Statistics and Bioinformatics, Medical Faculty, University of Belgrade, Belgrade, Serbia; 4 University Clinical Hospital Center “Zvezdara”, Department for Hemato-Oncology, Belgrade, Serbia; 5 Institute of Pathology, Medical Faculty, University of Belgrade, Belgrade, Serbia; 6 Department of Hematology, University Clinical Center of Podgorica, Medical Faculty, University of Podgorica, Podgorica, Montenegro

**Keywords:** 1q21 chromosomal abnormalities, multiple myeloma, prognosis, risk stratification, treatment

The reference for Reference 5 was erroneously written as “Shaughnessy J, Zhan F, Burington B, Huang Y, Colla S, Hanamura I, et al. Amplification and overexpression of CKS1B at chromosome band 1q21 is associated with reduced p27Kip1 and an aggressive clinical course in MM. *Hematology*. (2005) 10(Suppl 1):117–126. doi: 10.1182/blood-2005-03-1036”.

It should be “Shaughnessy J. Amplification and overexpression of CKS1B at chromosome band 1q21 is associated with reduced levels of p27Kip1 and an aggressive clinical course in multiple myeloma. *Hematology*. 2005;10 Suppl 1:117-26. doi: 10.1080/10245330512331390140”.

The reference for Reference 20 was erroneously written as “Walker BA, Wardell CP, Melchor L, Brioli A, Johnson DC, Kaiser MF, et al. Intraclonal heterogeneity and clonal evolution in myeloma. *Blood*. (2014) 124:1057–66. doi: 10.1182/blood-2014-03-560011”.

It should be “Walker BA, Wardell CP, Melchor L, Brioli A, Johnson DC, Kaiser MF, et al. Intraclonal heterogeneity is a critical early event in the development of myeloma and precedes the development of clinical symptoms. *Leukemia*. 2014 Feb;28(2):384-390. doi: 10.1038/leu.2013.199”.

The reference for Reference 21 was erroneously written as “Bolli N, Avet-Loiseau H, Wedge DC, Van Loo P, Alexandrov LB, Martincorena I, et al. Genomic evolution in multiple myeloma. *Nat Commun*. 2018;9:3363”.

It should be “Bolli N, Avet-Loiseau H, Wedge DC, Van Loo P, Alexandrov LB, Martincorena I, et al. Heterogeneity of genomic evolution and mutational profiles in multiple myeloma. *Nat Commun*. 2014;5:2997. doi: 10.1038/ncomms3997”.

The reference for Reference 22 was erroneously written as “Neben K, Hielscher T, Jauch A, Hillengass J, Raab MS, Ho AD, et al. Complex chromosomal aberrations in multiple myeloma: a German-speaking myeloma multicenter study. *Blood*. (2013) 122:3226–35. doi: 10.1182/blood-2013-03-491209”.

It should be “Neben K, Jauch A, Bertsch U, Heiss C, Hielscher T, Seckinger A, et al. Combining information regarding chromosomal aberrations t(4;14) and del(17p13) with the International Staging System classification allows stratification of myeloma patients undergoing autologous stem cell transplantation. *Haematologica*. 2010 Jul;95(7):1150-7. doi: 10.3324/haematol.2009.016436”.

The reference for Reference 23 was erroneously written as “Weinhold N, Salwender H, Heiß C, Neben K, Goldschmidt H, Bertsch U, et al., 1q21 abnormalities and prognosis in multiple myeloma. *Leukemia*. (2016) 30:2023–2031. doi: 10.1038/leu.2016.116”.

It should be “Weinhold N, Kirn D, Seckinger A, Hielscher T, Granzow M, Bertsch U, et al. Concomitant gain of 1q21 and MYC translocation define a poor prognostic subgroup of hyperdiploid multiple myeloma. *Haematologica*. (2016) 101(3):e116–e119. doi: 10.3324/haematol.2015.136929”.

The reference for Reference 24 was erroneously written as “Kastritis E, Migkou M, Dalampira D, Kanellias N, Eleutherakis-Papaiakovou E, Gavriatopoulou M, et al. Ultra–high-risk myeloma. *Am J Hematol*. 2022;97(12):1615–1626”.

It should be “Kastritis E, Migkou M, Dalampira D, Gavriatopoulou M, Fotiou D, Roussou M, et al. Chromosome 1q21 aberrations identify ultra high-risk myeloma with prognostic and clinical implications. *Am J Hematol*. 2022 Sep;97(9):1142-1149. doi: 10.1002/ajh.26639”.

The reference for Reference 27 was erroneously written as “Sonneveld P, Schmidt-Wolf IGH, van der Holt B, El Jarari L, Bertsch U, Salwender H, et al. HOVON-65/GMMG-HD4 trial. *J Clin Oncol*. (2012) 30:2946–55. doi: 10.1200/JCO.2011.39.6820”.

It should be “Sonneveld P, Schmidt-Wolf IG, van der Holt B, El Jarari L, Bertsch U, Salwender H, et al. Bortezomib induction and maintenance treatment in patients with newly diagnosed multiple myeloma: results of the randomized phase III HOVON-65/GMMG-HD4 trial. *J Clin Oncol*. 2012 Aug 20;30(24):2946-55. doi: 10.1200/JCO.2011.39.6820”.

The reference for Reference 30 was erroneously written as “Kumar SK, Mikhael JR, Buadi FK, Dingli D, Dispenzieri A, Fonseca R, et al. Mayo cytogenetic classification. *Mayo Clin Proc*. (2017) 92:578–88. doi: 10.1016/j.mayocp.2017.01.003”.

It should be “Dingli D, Ailawadhi S, Bergsagel PL, Buadi FK, Dispenzieri A, Fonseca R, et al. Therapy for Relapsed Multiple Myeloma: Guidelines From the Mayo Stratification for Myeloma and Risk-Adapted Therapy. *Mayo Clin Proc*. 2017 Apr;92(4):578-598. doi:10.1016/j.mayocp.2017.01.003”.

The reference for Reference 31 was erroneously written as “Attal M, Lauwers-Cances V, Hulin C, Leleu X, Caillot D, Escoffre M, et al. IFM/DFCI 2009 trial. *N Engl J Med*. (2017) 376:1311–20. doi: 10.1056/NEJMoa1611750”.

It should be “Attal M, Lauwers-Cances V, Hulin C, Leleu X, Caillot D, Escoffre M, et al. IFM 2009 Study. Lenalidomide, Bortezomib, and Dexamethasone with Transplantation for Myeloma. *N Engl J Med*. 2017 Apr 6;376(14):1311-1320. doi: 10.1056/NEJMoa1611750”.

The reference for Reference 32 was erroneously written as “Gay F, Oliva S, Petrucci MT, Conticello C, Catalano L, Corradini P, et al. EMN02/HO95 trial. *Lancet Haematol*. (2019) 6:e534–46. doi: 10.1016/S2352-3026(19)30149-0”.

It should be “Cavo M, Gay F, Beksac M, Pantani L, Petrucci MT, Dimopoulos MA, et al. Autologous haematopoietic stem-cell transplantation versus bortezomib-melphalan-prednisone, with or without bortezomib-lenalidomide-dexamethasone consolidation therapy, and lenalidomide maintenance for newly diagnosed multiple myeloma (EMN02/HO95): a multicentre, randomised, open-label, phase 3 study. *Lancet Haematol*. 2020 Jun;7(6):e456-e468. doi: 10.1016/S2352-3026(20)30099-5”.

The referenec for Reference 33 was erroneously written as “Usmani SZ, Anderson KC, Dimopoulos MA, Chari A, Lonial S, Hajek R, et al. IMWG high-risk consensus. *Lancet Oncol*. (2024) 43:2739–51. doi: 10.1200/JCO.24.01062”.

It should be “Avet-Loiseau H, Davies FE, Samur MK, Corre J, D’Agostino M, Kaiser MF, et al. International Myeloma Society/International Myeloma Working Group Consensus Recommendations on the Definition of High-Risk Multiple Myeloma. *J Clin Oncol*. 2025 Aug 20;43(24):2739-2751. doi: 10.1200/JCO-24-01893”.

The referenec for Reference 35 was erroneously written as “Gay F, Musto P, Rota-Scalabrini D, Bertamini L, Belotti A, Galli M, et al. Carfilzomib-based induction and consolidation therapy with or without autologous stem-cell transplantation in patients with newly diagnosed multiple myeloma (FORTE): a randomised, open-label, phase 2 trial. *Lancet*. (2020) 396:186–97. doi:10.1016/S0140-6736(20)31726-6”.

It should be “Gay F, Musto P, Rota-Scalabrini D, Bertamini L, Belotti A, Galli M, et al. Carfilzomib with cyclophosphamide and dexamethasone or lenalidomide and dexamethasone plus autologous transplantation or carfilzomib plus lenalidomide and dexamethasone, followed by maintenance with carfilzomib plus lenalidomide or lenalidomide alone for patients with newly diagnosed multiple myeloma (FORTE): a randomised, open-label, phase 2 trial. *Lancet Oncol*. 2021 Dec;22(12):1705-1720. doi: 10.1016/S1470-2045(21)00535-0”.

The reference for Reference 36 was erroneously written as “Hari P, Zhang MJ, Roy V, Pérez WS, Bashey A, To LB, et al. Is the benefit of autologous stem cell transplantation maintained in multiple myeloma patients with high-risk cytogenetics? *Biol Blood Marrow Transplant*. (2019) 25:e83–91. doi: 10.1016/j.bbmt.2018.10.024”.

It should be “Zeng T, Zhou L, Xi H, Fu W, Du J, Zhang C, et al. Multiple myeloma patients at various cytogenetic risks benefit differently from autologous stem cell transplantation as a consolidation therapy. *Stem Cells Int*. 2015;2015:613045. doi: 10.1155/2015/613045”.

The reference for Reference 37 was erroneously written as “Mateos MV, Dimopoulos MA, Cavo M, Suzuki K, Jakubowiak A, Knop S, et al. Daratumumab plus lenalidomide and dexamethasone for untreated myeloma. *N Engl J Med*. (2019) 380:2104–15. doi: 10.1056/NEJMoa1817249”.

It should be “Facon T, Kumar S, Plesner T, Orlowski RZ, Moreau P, Bahlis N, et al. MAIA Trial Investigators. Daratumumab plus Lenalidomide and Dexamethasone for Untreated Myeloma. *N Engl J Med*. 2019 May 30;380(22):2104-2115. doi: 10.1056/NEJMoa1817249”.

The original version of this article has been updated.

