# Supplementary material for: Multiple myeloma in the real world settings: prognostic significance of 1q21 chromosomal abnormalities - single center experience
Source: Front Oncol. 2026 Feb 11;16:1765235. doi: 10.3389/fonc.2026.1765235 (PMC12932195; doi:10.3389/fonc.2026.1765235)
Supplement: Supplementary file 1 [file Table1.docx]

Supplementary Material

# Supplementary Figures and Tables

**Table 1. Distribution of patients in accordance with demographic, laboratory characteristics and staging systems.**

| **Gender - no (%)** | |
| --- | --- |
| Male | 163 (49.7%) |
| Female | 165 (50.3%) |
| **Multiple myeloma type - no (%)** | |
| IgG | 204 (62.2%) |
| IgA | 69 (21%) |
| Bence-Jonce (BJ) | 54 (16.5%) |
| IgD | 1 (0.3%) |
| **Clinical Stage (Durie & Salmon) - no (%)** | |
| CS I | 24 (7.3%) |
| CS II | 28 (8.6%) |
| CS III | 276 (84.1%) |
| Renal impairment (B) | 78 (23.9%) |
| **ISS score - no (%)** | |
| ISS 1 | 99 (30.2%) |
| ISS 2 | 88 (26.8%) |
| ISS 3 | 141 (43%) |
| **R-ISS score - no (%)** | |
| R-ISS 1 | 92 (28%) |
| R-ISS 2 | 201 (61.3%) |
| R-ISS 3 | 35 (10.7%) |
| **R2-ISS score - no (%)** | |
| R2-ISS 1 | 84 (25.6%) |
| R2-ISS 2 | 75 (22.9%) |
| R2-ISS 3 | 152 (46.3%) |
| R2-ISS 4 | 17 (5.2%) |

**Table 2. iFISH findings in analyzed group of MM patients**

| **Chromosomal abnormalities** | **Pts, no (%)** |
| --- | --- |
| abnormalities of 1q21 | 76 (33.7%) |
| amp1q21 | 48 (21.3%) |
| gain 1q21 | 28 (12.4%) |
| del 17p13 | 30 (10.6%) |
| del1p32 | 22 (7.7%) |
| t(4;14) | 32 (14.2%) |
| t(14;16) | 8 (2.8%) |
| t(11;14) | 39 (17.3%) |
| Double and Triple Hit MM | 16 (5.6%) |

**Table 3. Summarized factors with influence on PFS and OS by multivariate model**

| **PFS Covariates** | **Hazard ratio, 95% CI** | ***p* value** |
| --- | --- | --- |
| ASCT eligibility | 0.39 (0.20-0.64) | *p*<0.01 |
| ISS | 1.39 (1.08-1.79) | *p*<0.01 |
| t(14;16) | 3.47 (1.18-10.2) | *p*=0.023 |
| amp1q21 | 1.68 (0.98-2.88) | *p*=0.058 |
| **OS Covariates** | **Hazard ratio, 95% CI** | ***p* value** |
| ASCT eligibility | 0.17 (0.08-0.35) | *p*<0.01 |
| ISS | 1.7 (1.26-2.30) | *p*<0.01 |
| Age >65yrs | 0.6 (0.39-0.95) | *p*=0.029 |
